# A Prematurity Collaborative Birth Equity Consensus Statement for Mothers and Babies

**DOI:** 10.1007/s10995-020-02960-0

**Published:** 2020-06-16

**Authors:** Fleda Mask Jackson, Kweli Rashied-Henry, Paula Braveman, Tyan Parker Dominguez, Diana Ramos, Noble Maseru, William Darity, Lisa Waddell, Donald Warne, Gina Legaz, Rahul Gupta, Arthur James

**Affiliations:** 1 Save 100 Babies and MAJAICA, LLC, Atlanta, GA USA; 2 March of Dimes, Arlington, VA USA; 3 Center on Social Disparities in Health, University of California, San Francisco, San Francisco, CA USA; 4 USC Suzanne Dworak-Peck School of Social Work, Los Angeles, CA USA; 5 Keck School of Medicine, University of Southern California, Los Angeles, CA USA; 6 Department of Behavioral and Community Health Sciences, University of Pittsburgh Graduate School of Public Health, Pittsburgh, PA USA; 7 Sanford School of Public Policy, Department of African and African American Studies, Department of Economics, Duke University, Durham, NC USA; 8 School of Medicine & Health Sciences, University of North Dakota, Grand Forks, ND USA; 9 Wexner Medical Center, The Ohio State University (Emeritus), Columbus, OH USA

**Keywords:** Birth equity, Health equity, Equity, Preterm birth, Social determinants, Social sciences, Maternal

## Abstract

**Introduction:**

In 2016, March of Dimes (MOD) launched its Prematurity Collaborative to engage a broad cross section of national experts to address persistent and widening racial disparities in preterm birth by achieving equity and demonstrated improvements in preterm birth. African-American and Native American women continue to have disproportionate rates of preterm birth and maternal death. As part of the Collaborative, MOD created the Health Equity Workgroup whose task was the creation of a scientific consensus statement articulating core values and a call to action to achieve equity in preterm birth utilizing health equity and social determinants of health frameworks.

**Methods:**

Health Equity Workgroup members engaged in-person and virtually to discuss key determinant contributors and resolutions for disparate maternal and birth outcomes. Workgroup members then drafted the Birth Equity Consensus Statement that contained value statements and a call to action. The birth equity consensus statement was presented at professional conferences to seek broader support. This article highlights the background and context towards arriving at the core values and call to action, which are the two major components of the consensus statement and presents the core values and call to action themselves.

**Results:**

The result was the creation of a birth equity consensus statement that highlights risks and protections of social determinants based on the prevailing science, and identifies promising solutions for reducing preterm birth and eliminating racial disparities.

**Conclusion:**

The birth equity consensus statement provides a mandate, guiding the work of March of Dimes and the broader MCH community, for equity-based research, practice, and policy advocacy at local, state, and federal levels.

**Significance:**

This field report adds to the current knowledge base on racial and ethnic disparities in birth and maternal health outcomes. Research has documented the science behind eliminating health disparities. Scientists and practitioners should continue to explore in practice how the social determinants of birth and maternal health, which manifest historically and contemporarily, can be addressed.

## Introduction

Building upon its past successes in eradicating polio, March of Dimes (MOD) shifted its focus in 2003 to prematurity, with the goal of decreasing the preterm birth rate by at least 15%. Between 2015 and 2017, the U.S. preterm birth rate increased from 9.6 to 9.8%. Preterm birth rates were 50% higher among non-Hispanic black/African-American women and 20% higher among American Indian/Alaska Native women compared to non-Hispanic white women (March of Dimes 2018). African-American and Native American women continue to have disproportionate rates of preterm birth and maternal death. (Note: In this statement the terms “black” and “African-American,” and “American-Indian/Alaskan Native” and “Native American” are used interchangeably.)

Maternal death rates have also increased and are 3 to 4 times higher for black women than white women (Building U.S. Capacity to Review and Prevent Maternal Deaths 2018). The United States is the only industrialized nation that has seen a rise in the maternal death rate, even as rates in other countries have declined. Recent figures reveal 700 women die each year from pregnancy-related complications (Building U.S. Capacity to Review and Prevent Maternal Deaths 2018).

Complex explanations exist for adverse birth and maternal outcomes; nonetheless, scientific evidence indicates that unequal health care, socio-economic and racial inequalities pose significant health risks to pregnant women and their babies (Solar and Irwin 2010; WHO 2017; Smedley et al. 2003). In 2016, March of Dimes launched its Prematurity Collaborative to engage a broad cross section of national experts to address persistent and widening racial disparities in preterm birth by achieving equity and demonstrated improvements in preterm birth. The Collaborative, co-chaired by MOD and the Centers for Disease Control and Prevention (CDC), consisted of several work groups: Access to Care, Policy, and Health Equity. In 2017, the Health Equity Workgroup was established to guide the work of all Collaborative members.

The Health Equity Workgroup expanded its focus to include the health of mothers, recognizing that strategies used to reduce premature birth also support and promote maternal health. This work was informed by scientific research; the Collaborative’s guiding principles on equity; the World Health Organization’s SDOH framework; and the United Nations human rights declaration (Glendon 2001; World Health Organization 2017).

As part of the Collaborative, March of Dimes created the Health Equity Workgroup whose task was the creation of a scientific consensus statement articulating core values and a call to action to achieve equity in preterm birth utilizing health equity and social determinants of health (SDOH) frameworks.

The objectives for the Health Equity Workgroup were to:

- Advance SDOH as the primary basis for disparities in maternal and birth outcomes;
- Articulate and advance core birth equity values to guide the work required to achieve the best possible maternal and birth outcomes for all mothers and babies;
- Create a call to action with equity-focused recommendations for work across and beyond the Collaborative, based upon the core values and the current science.

This document presents the work of the Health Equity Workgroup that resulted in a birth equity consensus statement on the key drivers of prematurity and maternal mortality disparities to prompt transformation and advancements in research, policy, and advocacy.

## Methods

The MOD Prematurity Collaborative Health Equity Workgroup is comprised of approximately 300 individuals who represent a cross-section of partners committed to maternal and child health. Members were invited or requested to join. Quarterly meetings were facilitated in-person and virtually and offered insight into best practices for reducing racial and ethnic disparities in birth outcomes. The initial effort of the Workgroup was to produce a set of guiding principles and glossary to achieve equity in preterm birth. Workgroup members discussed key determinant contributors and resolutions for disparate maternal and birth outcomes and convened to write the birth equity consensus statement to reinforce inquiry and action by the social sciences based on the equity and SDOH perspectives. Workgroup members then drafted the Birth Equity Consensus Statement that contained value statements and a call to action. The birth equity consensus statement was presented at professional conferences to seek broader support. This article specifically highlights the background and context towards arriving at the core values and call to action, which are the two major components of the consensus statement and presents the core values and call to action themselves.

This work was conducted in accord with prevailing ethical principles and does not constitute human subjects research. The manuscript is not based upon clinical study or patient data.

In the review of the literature we identified the following core areas: health care access, psychosocial conditions and economic inequality that were of interest to the Health Equity Workgroup participants, which led to generating a consensus agreement process of the Collaborative. The following sections provide support for the consensus statement components.

Equity is a human right built upon the belief that all individuals are of equal worth and should be afforded respect, dignity, justice, and fairness. When applied to health, health equity means that all human beings must have every opportunity that is fair and just to achieve optimal health (Braveman et al. 2018). According to the CDC, health equity is when everyone has the opportunity to be as healthy as possible (CDC 2020). By extension, birth equity is “the assurance of the conditions of optimal births for all people with a willingness to address racial and social inequalities in a sustained effort.” (National Birth Equity Collaborative 2019).

MOD’s creation of a birth equity consensus statement emphasizing SDOH aligns with an expanding approach within the broader MCH community. This direction highlights SDOH as an explanation for racial disparities alongside medical and biological causes. While discourse persists about SDOH versus medical/biological explanations for racial and ethnic disparities, evidence points to SDOH playing a significant role in birth and maternal health outcomes (Solar and Irwin 2010).

While not explicitly applied to maternal and birth outcomes, the work of President and MOD founder, Theodore Roosevelt and First Lady Eleanor Roosevelt, director of the United Nations Human Rights agenda, foretold the significance of equity and SDOH. The Prematurity Collaborative’s creation of a birth equity consensus document draws, in part, upon past MOD initiatives, the social reform legislative agenda of President Roosevelt and the human rights agenda led by Eleanor Roosevelt.

Through MOD early support for development and dissemination of a polio vaccine for all children, polio declined drastically to almost virtual elimination. The initial work of MOD, however, was not without controversy. Black civil rights leaders, journalists, sororities, and health professionals challenged the organization’s early acquiescence to segregation and discrimination, which limited access to polio care and resources for blacks especially in the Deep South (Mawdsky 2010). Their efforts led MOD to hire Charles Bynum, in 1944, to champion the inclusion of African-Americans in all aspects of the national polio eradication campaign and treatment centers during the height of the epidemic (Mawdsky 2010). The work of the Prematurity Collaborative builds upon the premise of equality advanced by President Roosevelt and the First Lady to uphold equity and its requirement for specific resources to address the key drivers of disparities for black and Native American women.

### Health Care Access

In 2016, the U.S. spent nearly twice as much on health care as 10 other high-income countries (Papanicolas et al. 2018), yet its infant and maternal mortality outcomes were among the worst. The reasons for poorer overall U.S. health outcomes are multifaceted; however, subpar health care is a key factor (Howell et al. 2016). Less accessible health care due to affordability and availability within communities of poor women and women of color who are at the highest risk impedes improved maternal health and pregnancy outcomes (Prather et al. 2018).

Besides access, unequal treatment in the health care system, stemming from implicit or explicit bias is a significant health risk for pregnant women and their babies (Smedley et al. 2003). Institutionalized racism in health care has a long history of denying women of color the best possible pregnancy and maternal care. These injustices can be traced back to the beginnings of modern gynecological practices. For example, in the 1800s as scientific racism emerged, enslaved black women were brutally experimented upon without anesthetics to develop gynecological procedures (Roberts 1997). The long-held belief that blacks have a higher tolerance for pain continues to be a basis for denying black women equitable health care (Hoffman et al. 2016). Similarly, Native American women have a history of forced sterilization as part of reproductive health care received at Indian Health Services; and controversy continues around the testing of high dose hormonal contraceptives with Puerto Rican women (Lawrence 2000; Liao and Dollin 2012).

Recent investigations suggest that while interventions are available to inhibit premature birth and prevent adverse maternal outcomes, black women and other women of color have not had equal access to these treatments (Rowley and Hogan 2012). The marginalization of poor women and women of color by health care systems, along with the history of scientific misconduct in the care of African-American, Latina and Native American women, has created distrust. Continued skepticism and distrust are not unwarranted as sub-standard quality of care is often commonplace in health care systems that serve under-resourced communities.

Inequity in maternity care provides only a partial explanation for the rising rates and persistent disparities in preterm birth and maternal mortality. SDOH—the socio-environmental exposures connected to the places where individuals are born, grow, live, age, work and play–exert a major influence on health outcomes. Research connecting health outcomes to SDOH illustrates the significance of equitable policies on housing, education, and childcare (Din-Dzietham and Hertz-Picciotto 1998; Polednak 1996). While 60% of poor health can be attributed to factors outside of health care, when it comes to spending on social services, the U.S. ranks in the bottom 10 among developed countries (Freedman 2018).

### Psychosocial Conditions

The significance of racial segregation in the context of increased rates of preterm birth has relevance for social policies. Well before public health and medical communities studied the ill health effects of racial inequities in segregated neighborhoods, the work of Kenneth and Mamie Clark foretold the current research connecting place-based inequalities to disparate physical and mental health outcomes (Clark and Clark 1950). Their work demonstrating the damaging effects of racism on the psychological growth and development of black children became the scientific basis for the passage of the most significant decision on racial equality in the twentieth century, Brown v. Board of Education in 1954.

Non-medical factors can help explain persistent disparities in birth outcomes. College-educated black women have worse birth outcomes than their white college-educated counterparts (Din-Dzietham and Hertz-Picciotto 1998). They also fare worse in comparison to white women who are less educated, unemployed, and uninsured. Studies exploring a solely genetic explanation for consistent racially disparate birth outcomes remain unproven, inconclusive, and unproductive towards dispelling false narratives of the origins of race as a biologic construct (David and Collins 2007).

Intersectionality refers to the convergence of multiple identities and forms of discrimination (Jackson et al. 2001). When appropriated within a birth equity context, it reveals how racism and sexism simultaneously produce emotional and physiological responses that can lead to poor birth outcomes (Nuru-Jeter et al. 2009). Evidence shows a significant association between lower birthweight and racial discrimination experienced by black women (Dominguez et al. 2008). Research also shows the link between stress resulting from gendered racism and antenatal depression that places both mother and infant in jeopardy (Jackson et al. 2012).

The risk for poor birth outcomes as a consequence of SDOH begins long before pregnancy and childbirth. The accumulation of environmental exposures, starting in utero, may determine differential risks and protection for health outcomes across the life course (Shonkoff et al. 2012). In the case of pregnancy and inequity, studies suggest that repeated stress that accumulates over time triggers responses leading to early birth (Jackson et al. 2012). The “weathering” over time, from constant assaults from inequity places a woman at risk of an adverse pregnancy outcome (Geronimus 1992), conferring risk upon her infant for compromised health throughout the life course (Barker 2012). The study of epigenetics builds on these associations by exploring gene expression based on environmental changes and considers the multi-generational impact of these biological changes (NASEM 2019). Further examination in this growing area is needed.

### Economic Inequality

Economic inequality in the U.S. is “a product of historical and present-day forms of racism: labor, housing and other policies and practices…” (Kraus et al. 2017). Despite racial progress, racially based economic inequality contributes to poor health, including poor birth outcomes. While poverty certainly has an adverse impact on pregnancy, surprisingly poorer birth outcomes among well-educated black women may be linked to stress from incongruence between class expectations and racial economic inequalities, including a significant wealth gap (David and Collins 2007).

Compared to $1 earned in this country by a white male, Latina women earn 54 cents, African-American women earn 63 cents, and white women earn 78 cents (Kraus et al. 2017). African-American families earn just $57.30 for every $100 in white households (Kraus et al. 2017). Data also show the considerable wealth gap between black and white families, where black families hold on to less than 7 cents for every $1 a white family possesses (AAUW 2019). Even when a white family is near poverty, it has $18,000 in median wealth; whereas, a black family near poverty has a median wealth of near $0 (Kraus et al. 2017; AAUW 2019).

Further exploration of how the wealth gap contributes to poorer birth outcomes for well-educated women is needed. Unemployment, low wages, incarceration, and social policies affecting black fatherhood also present major impediments to a family’s economic stability. As skillfully articulated in a policy brief (Paul et al. 2018), we must build up the transformational vision of President Roosevelt to take bold action for families, mothers, fathers, and children by providing decent and significant employment for all Americans (FDR Presidential Library and Museum 2019).

The following section includes the actual result of these findings, which led to the consensus statement that highlights risks and protections of SDOH based on the prevailing science and identifies promising solutions for reducing preterm birth and eliminating racial disparities.

## Results: Collaborative Core Belief Statements on Birth Equity

Health equity requires addressing equity not only in health care but in all human-made and modifiable determinants of health (DeSisto et al. 2018). Justice and fairness, as the foundations of equity, demand rectifying historic and current injustices. Injustices may occur among marginalized or excluded groups of people based on their multiple and intersectional identities and/or other characteristics closely linked with social exclusion, marginalization, and social disadvantage. Therefore,

1. We believe that health is a human right and, we expect health with quality, accessible and affordable health care for everyone that is supported by equitable conditions across all sectors representing SDOH (education, transportation, housing, etc.).
2. We believe that ALL mothers and babies, regardless of race, ethnicity, culture, language and national origin; poverty and socioeconomic status; gender identity and sexual orientation; disability; and region and place (urban and rural) must have every opportunity for optimal maternal and birth outcomes.
3. We embrace birth equity as a directive for confronting inaccessible and inadequate prenatal and maternal health care, while challenging the inequalities in the distribution of power, income, wealth and other related factors (housing, employment, transportation) that contribute to persistent, unfair conditions expectant mothers impacted by negative birth outcomes endure.
4. We believe that undoing historical and contemporary patterns of racial and gender discrimination is imperative for disrupting implicit and explicit bias, miseducation and exclusionary customs and practices that can have dire health consequences for expectant mothers and babies that permeate systems of care; practice and treatment; education and research; public policies; communications; and access to funding and resources.
5. We respect the authoritative knowledge and assets among affected women, families and communities and regard their intersectional perspectives as paramount for the conceptualization and conduct of equity-driven research and its translation into maternal, paternal and child health education, practices and policies that are just and fair.
6. We believe that pursuit of explanations for racially disparate birth and maternal outcomes must employ social science disciplines and methods to best examine social and economic disadvantages as well as community and population assets for maternal mental and physical health.
7. We believe that scientific examination of the biological, clinical, social and environmental contributors to prematurity and maternal mortality must be inclusive of an equity framework in order to accelerate and translate reproductive health research and medical breakthroughs into innovative and accessible health care practices and policies that are equitable and beneficial for ALL expectant mothers and their infants.
8. We believe that the experience, expertise and leadership of researchers, practitioners and policymakers from historically underrepresented communities should be prioritized for birth equity solutions, including the provision of commensurate funding levels to advance equity for moms and babies.

## Call to Action and Conclusion

March of Dimes must exhibit the same collective will and dedicate needed resources that were instrumental for ending the polio epidemic to achieve birth equity. Following approaches to expand social determinant pathways for research, policy, and practice to promote health equity and thereby eliminating racial disparities in maternal and birth outcomes, we recommend:

- Expanding the scope of research, including birth and maternal outcomes surveillance, to include the fundamental determinant drivers of population health.
- Advancing equity informed approaches for research and evaluation that are mindful of and responsive to the impact of inequity on every aspect (biologic, physiologic, genetic, sociological, economic, and psychological) of the lives and well-being of impacted women and children.
- Engaging in health systems reform, including re-educating providers to better serve high risk populations.
- Working to undo beliefs, perceptions and practices associated with racism and discrimination to ensure that every woman at risk receives the care and support they need.
- Empowering communities through inclusion, education, social activism, and advocacy with full participation in cross sector efforts to confront and change social policies that are detrimental to mothers, fathers, babies, families and communities, recognizing that positive discernible changes in SDOH are essential for birth equity.

The birth equity consensus statement provides a mandate, guiding the work of March of Dimes and the broader MCH community, for equity-based research, practice, and policy advocacy at local, state and federal levels. March of Dimes’ focus on SDOH supports the ongoing quest for birth equity for all mothers and babies.
